# Supplementary material for: Chronic hyperactivation of midbrain dopamine neurons causes preferential dopamine neuron degeneration
Source: bioRxiv. 2024 May 13:2024.04.05.588321. Originally published 2024 Apr 10. Preprint. [Version 2] doi: 10.1101/2024.04.05.588321 (PMC11030348; doi:10.1101/2024.04.05.588321)
Supplement: Supplementary file 1 [file media-1.pdf]

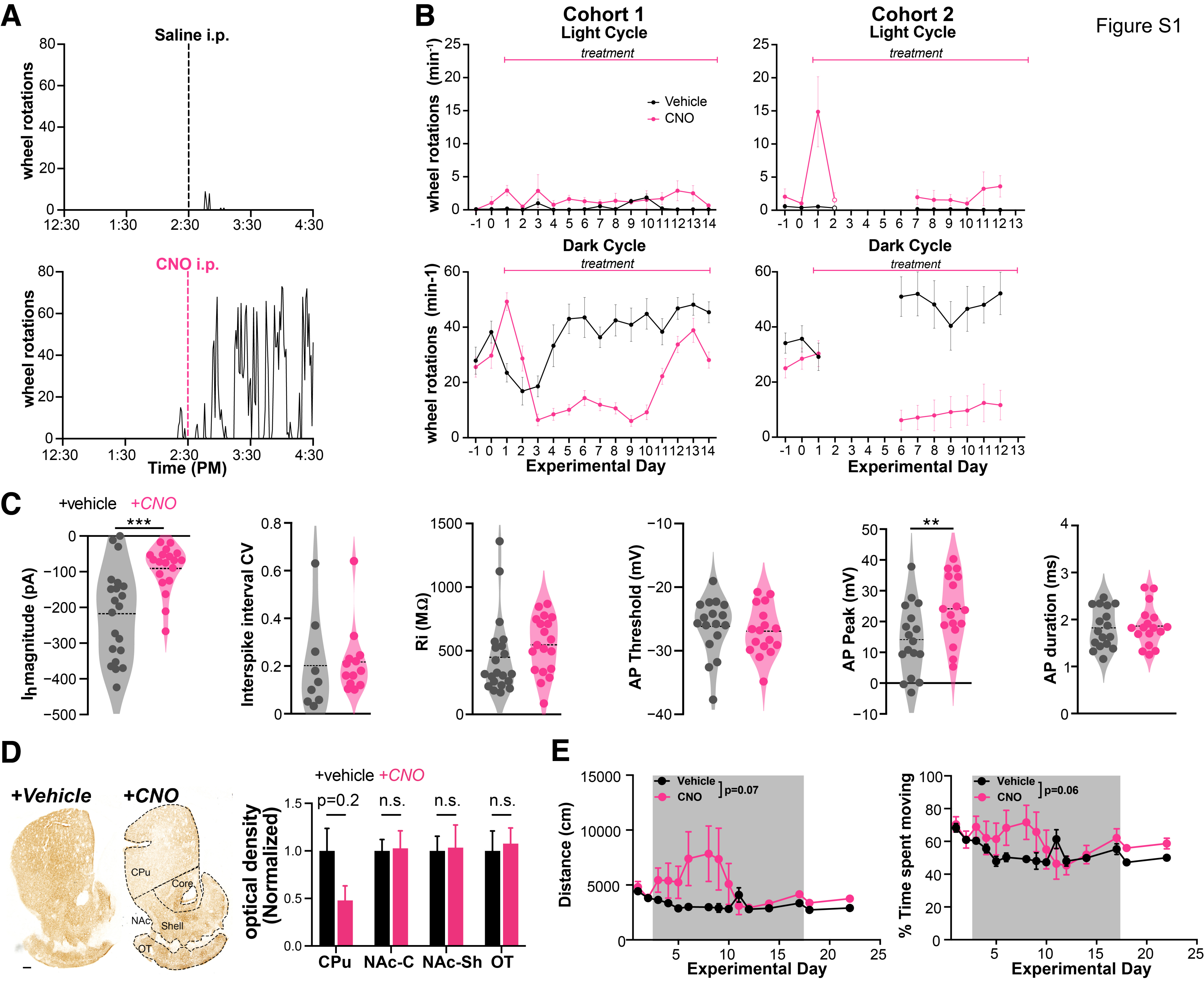
Figure S1
A
Saline i.p.
wheel rotations
CNO i.p.
Time (PM)
B
Cohort 1
Light Cycle
Cohort 2
Light Cycle
treatment
wheel rotations (min-1)
Vehicle
CNO
Dark Cycle
Experimental Day
C
+vehicle +CNO
***
Ih magnitude (pA)
Interspike interval CV
Ri (MΩ)
AP Threshold (mV)
**
AP Peak (mV)
AP duration (ms)
D
+Vehicle
+CNO
CPu
Core
NAc
Shell
OT
+vehicle +CNO
optical density (Normalized)
p=0.2
n.s.
CPu
NAc-C
NAc-Sh
OT
E
Vehicle
CNO
p=0.07
Distance (cm)
Experimental Day
p=0.06
% Time spent moving

Figure S2

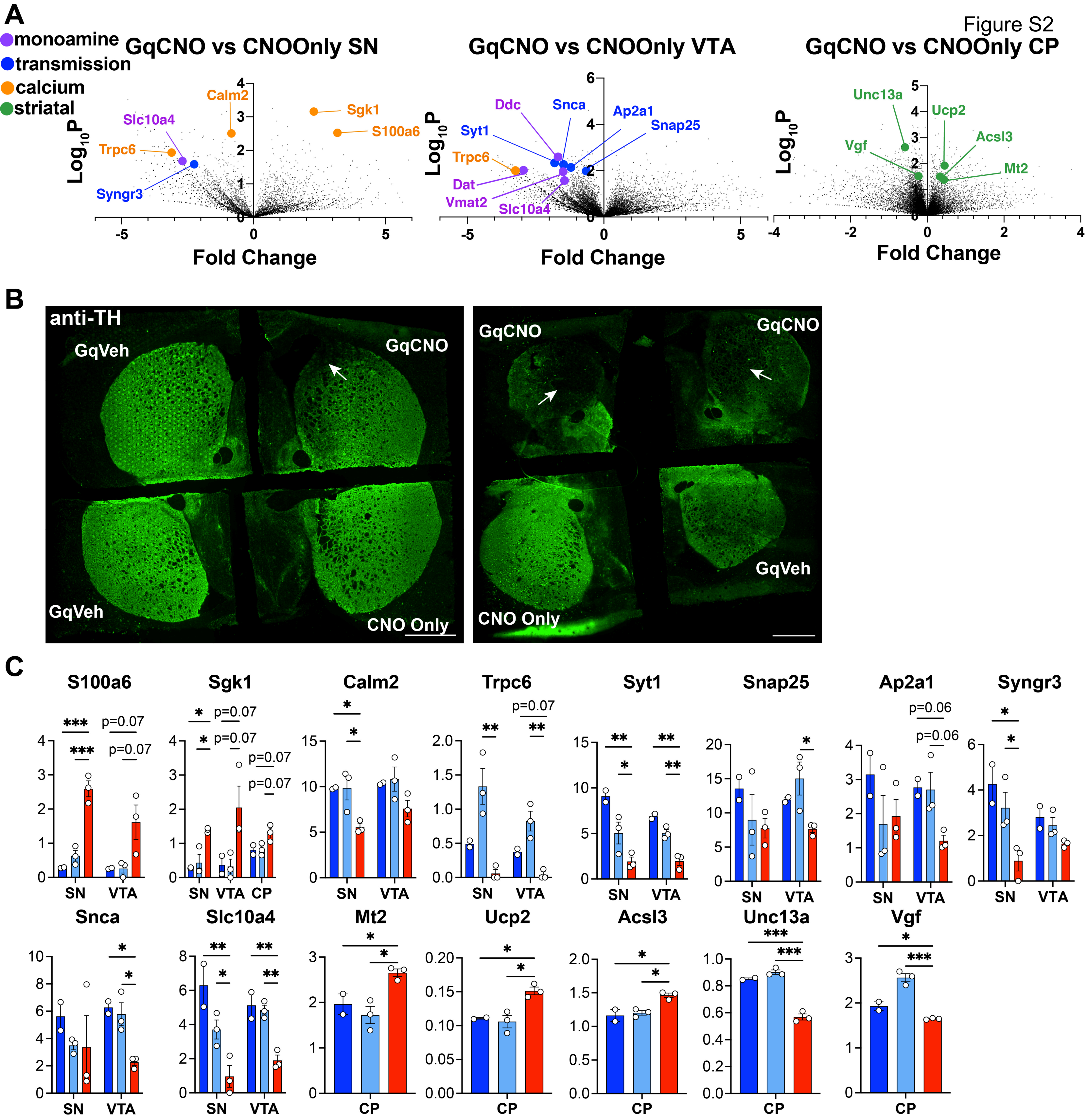
A
monoamine
transmission
calcium
striatal
GqCNO vs CNOOnly SN
GqCNO vs CNOOnly VTA
GqCNO vs CNOOnly CP
Calm2
Sgk1
S100a6
Slc10a4
Trpc6
Syngr3
Ddc
Snca
Ap2a1
Snap25
Syt1
Dat
Vmat2
Unc13a
Ucp2
Acsl3
Vgf
Mt2
Log10P
Fold Change
B
anti-TH
GqVeh
GqCNO
CNO Only
C
S100a6
Sgk1
Calm2
Trpc6
Syt1
Snap25
Ap2a1
Syngr3
Snca
Slc10a4
Mt2
Ucp2
Acsl3
Unc13a
Vgf
SN
VTA
CP
p=0.07
p=0.06

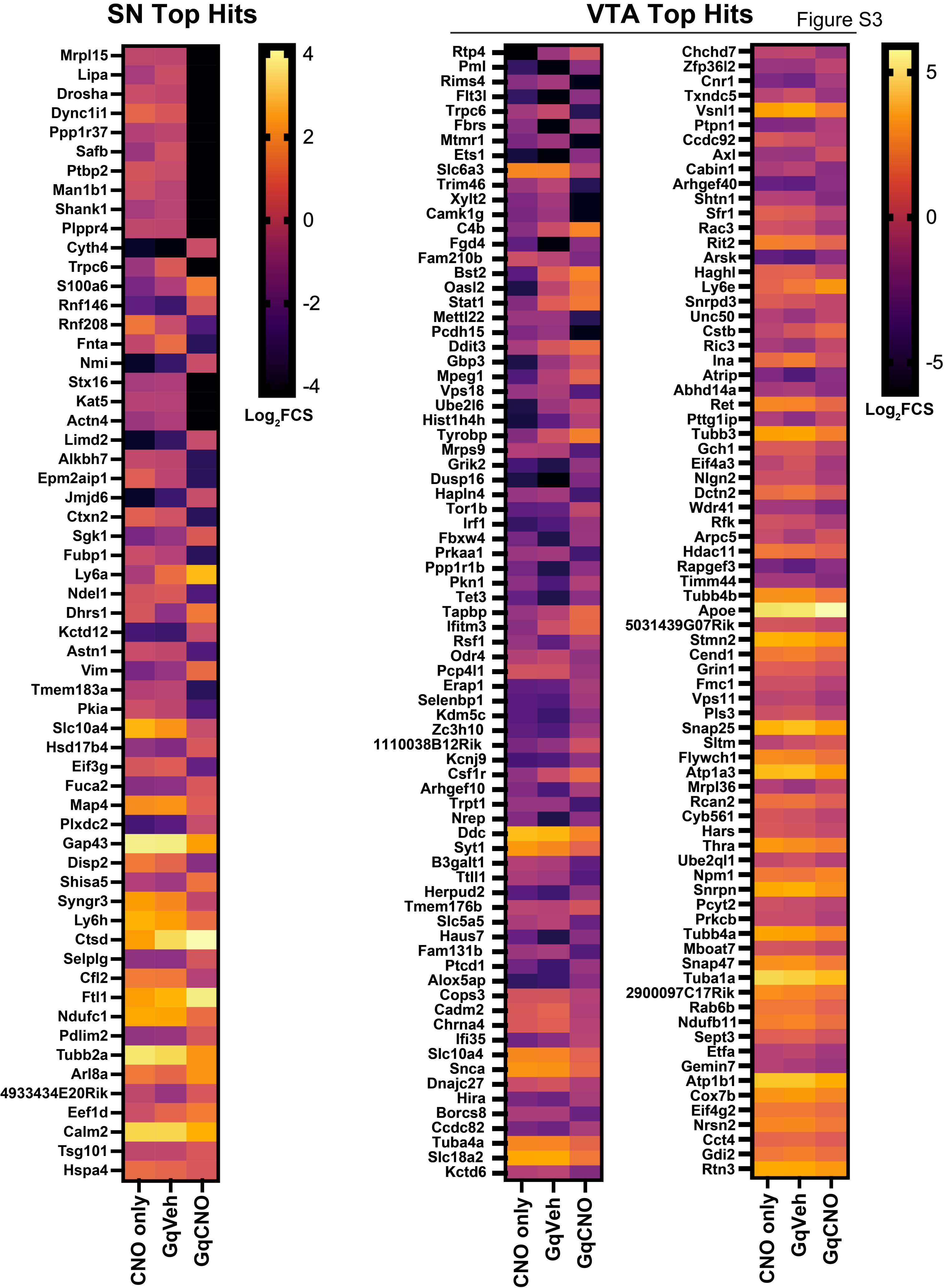
Figure S3
SN Top Hits
Mrpl15
Lipa
Drosha
Dync1i1
Ppp1r37
Safb
Ptbp2
Man1b1
Shank1
Plppr4
Cyth4
Trpc6
S100a6
Rnf146
Rnf208
Fnta
Nmi
Stx16
Kat5
Actn4
Limd2
Alkbh7
Epm2aip1
Jmjd6
Ctxn2
Sgk1
Fubp1
Ly6a
Ndel1
Dhrs1
Kctd12
Astn1
Vim
Tmem183a
Pkia
Slc10a4
Hsd17b4
Eif3g
Fuca2
Map4
Plxdc2
Gap43
Disp2
Shisa5
Syngr3
Ly6h
Ctsd
Selplg
Cfl2
Ftl1
Ndufc1
Pdlim2
Tubb2a
Arl8a
4933434E20Rik
Eef1d
Calm2
Tsg101
Hspa4
CNO only
GqVeh
GqCNO
4
2
0
-2
-4
Log2FCS
VTA Top Hits
Rtp4
Pml
Rims4
Flt3l
Trpc6
Fbrs
Mtmr1
Ets1
Slc6a3
Trim46
Xylt2
Camk1g
C4b
Fgd4
Fam210b
Bst2
Oasl2
Stat1
Mettl22
Pcdh15
Ddit3
Gbp3
Mpeg1
Vps18
Ube2l6
Hist1h4h
Tyrobp
Mrps9
Grik2
Dusp16
Hapln4
Tor1b
Irf1
Fbxw4
Prkaa1
Ppp1r1b
Pkn1
Tet3
Tapbp
Ifitm3
Rsf1
Odr4
Pcp4l1
Erap1
Selenbp1
Kdm5c
Zc3h10
1110038B12Rik
Kcnj9
Csf1r
Arhgef10
Trpt1
Nrep
Ddc
Syt1
B3galt1
Ttll1
Herpud2
Tmem176b
Slc5a5
Haus7
Fam131b
Ptcd1
Alox5ap
Cops3
Cadm2
Chrna4
Ifi35
Slc10a4
Snca
Dnajc27
Hira
Borcs8
Ccdc82
Tuba4a
Slc18a2
Kctd6
CNO only
GqVeh
GqCNO
Chchd7
Zfp36l2
Cnr1
Txndc5
Vsnl1
Ptpn1
Ccdc92
Axl
Cabin1
Arhgef40
Shtn1
Sfr1
Rac3
Rit2
Arsk
Haghl
Ly6e
Snrpd3
Unc50
Cstb
Ric3
Ina
Atrip
Abhd14a
Ret
Pttg1ip
Tubb3
Gch1
Eif4a3
Nlgn2
Dctn2
Wdr41
Rfk
Arpc5
Hdac11
Rapgef3
Timm44
Tubb4b
Apoe
5031439G07Rik
Stmn2
Cend1
Grin1
Fmc1
Vps11
Pls3
Snap25
Sltm
Flywch1
Atp1a3
Mrpl36
Rcan2
Cyb561
Hars
Thra
Ube2ql1
Npm1
Snrpn
Pcyt2
Prkcb
Tubb4a
Mboat7
Snap47
Tuba1a
2900097C17Rik
Rab6b
Ndufb11
Sept3
Etfa
Gemin7
Atp1b1
Cox7b
Eif4g2
Nrsn2
Cct4
Gdi2
Rtn3
CNO only
GqVeh
GqCNO
5
0
-5
Log2FCS

Figure S4

CP Top Hits

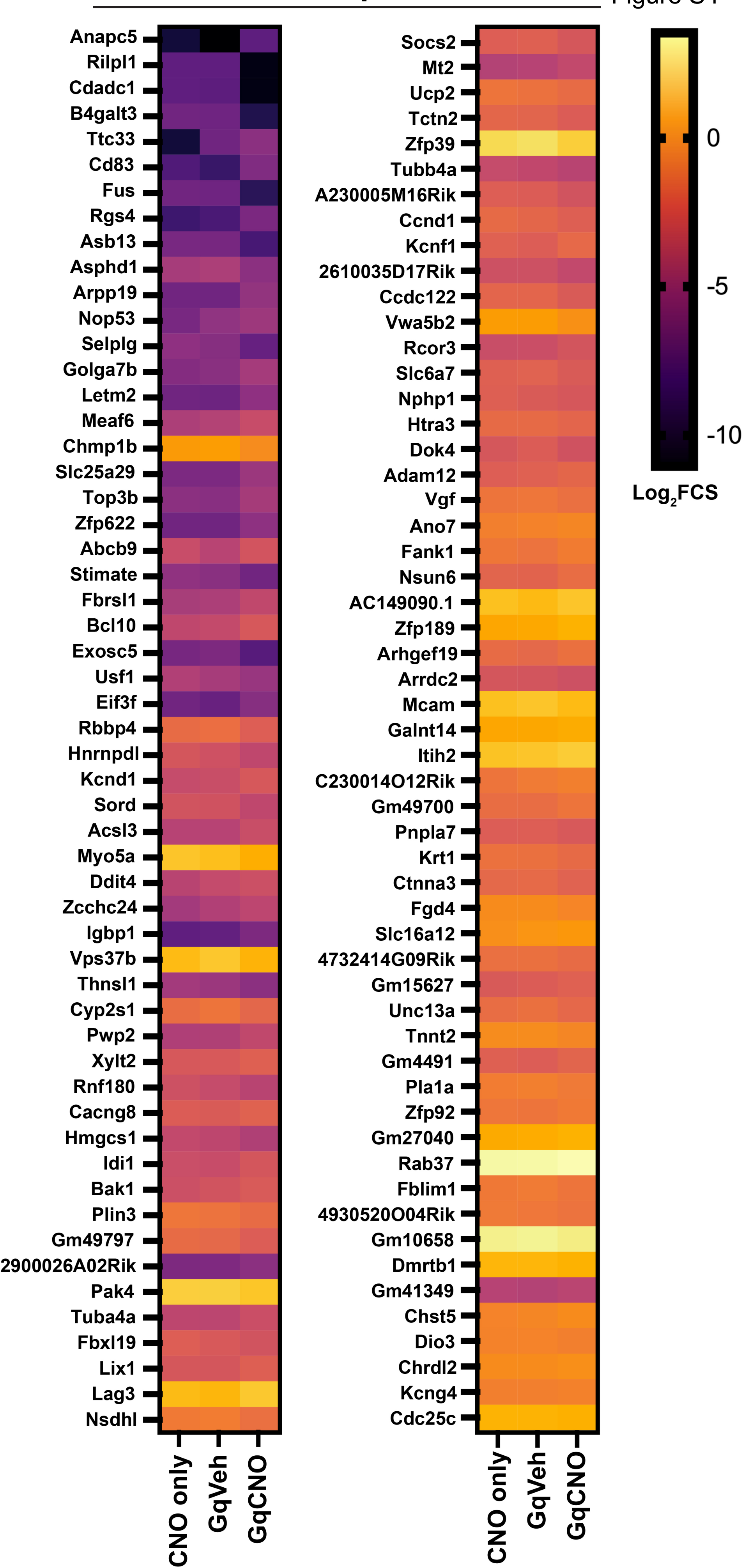

Figure S5

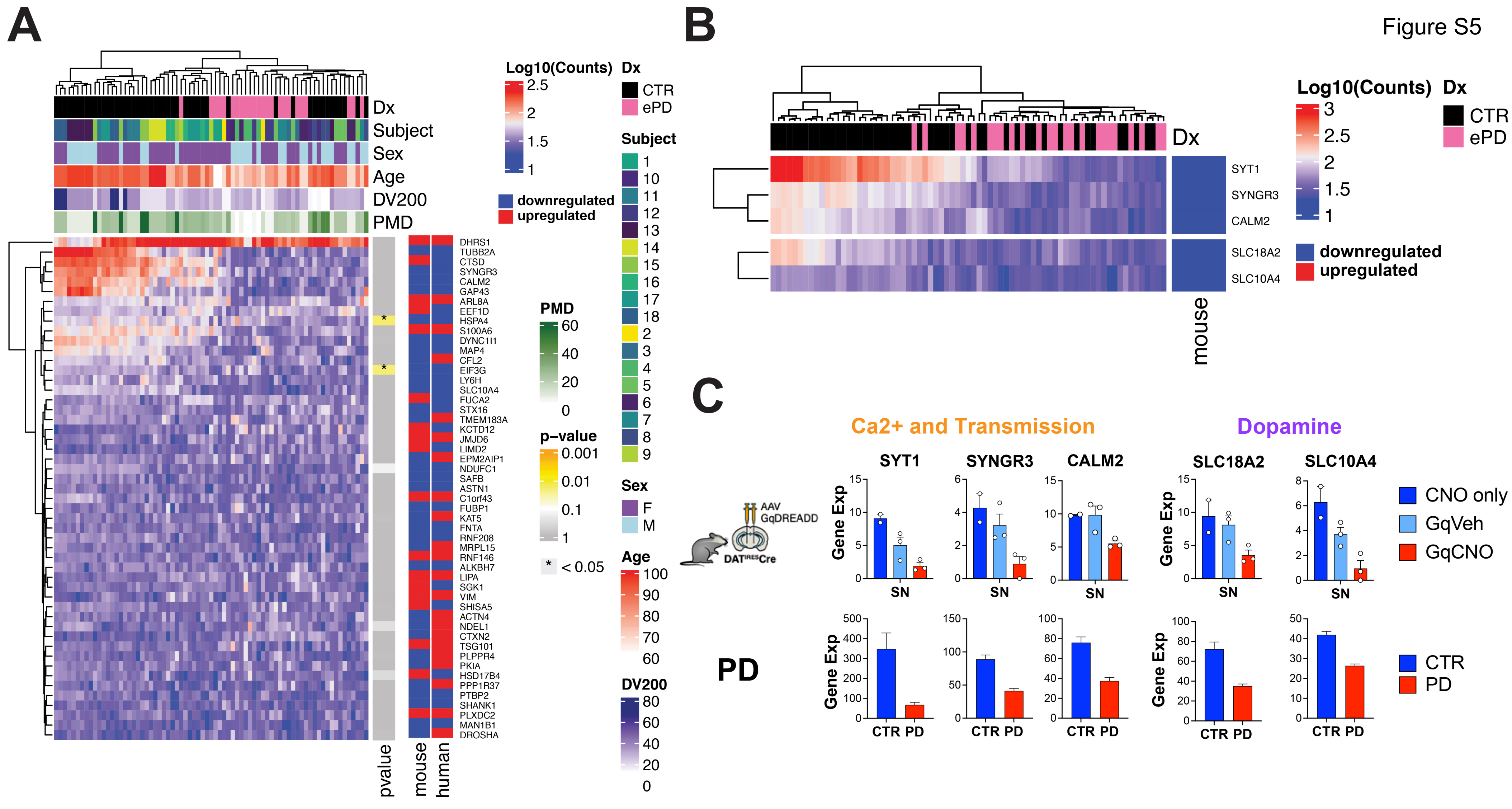
A
Dx
Subject
Sex
Age
DV200
PMD
Log10(Counts)
Dx
CTR
ePD
downregulated
upregulated
Subject
PMD
p–value
* < 0.05
Sex
F
M
Age
DV200
pvalue
mouse
human
DHRS1
TUBB2A
CTSD
SYNGR3
CALM2
GAP43
ARL8A
EEF1D
HSPA4
S100A6
DYNC1I1
MAP4
CFL2
EIF3G
LY6H
SLC10A4
FUCA2
STX16
TMEM183A
KCTD12
JMJD6
LIMD2
EPM2AIP1
NDUFC1
SAFB
ASTN1
C1orf43
FUBP1
KAT5
FNTA
RNF208
MRPL15
RNF146
ALKBH7
LIPA
SGK1
VIM
SHISA5
ACTN4
NDEL1
CTXN2
TSG101
PLPPR4
PKIA
HSD17B4
PPP1R37
PTBP2
SHANK1
PLXDC2
MAN1B1
DROSHA
B
Dx
SYT1
SYNGR3
CALM2
SLC18A2
SLC10A4
mouse
Log10(Counts)
Dx
CTR
ePD
downregulated
upregulated
C
Ca2+ and Transmission
Dopamine
AAV
GqDREADD
DAT^IRESCre
SYT1
SYNGR3
CALM2
SLC18A2
SLC10A4
Gene Exp
SN
CNO only
GqVeh
GqCNO
PD
CTR PD
CTR
PD

VTA

Index	GO Molecular Function 2023	P-value
1	GTP Binding (GO:0005525)	0.00002637
2	Syntaxin Binding (GO:0019905)	0.00005402
3	Guanyl Ribonucleotide Binding (GO:0032561)	0.00006568
4	Nuclear Receptor Coactivator Activity (GO:0030374)	0.0005911
5	Purine Ribonucleoside Triphosphate Binding (GO:0035639)	0.001174

Index	GO Biological Process 2023	P-value
1	Chemical Synaptic Transmission (GO:0007268)	0.00000141
2	Synaptic Vesicle Exocytosis (GO:0016079)	0.0000152
3	Anterograde Trans-Synaptic Signaling (GO:0098916)	0.00002437
4	Response To Cytokine (GO:0034097)	0.00005223
5	Response To Interferon-Beta (GO:0035456)	0.00006911

SN

Index	GO Molecular Function 2023	P-value
1	Phosphatase Activator Activity (GO:0019211)	0.001131
2	Tubulin Binding (GO:0015631)	0.002579
3	Calcium Channel Regulator Activity (GO:0005246)	0.005332
4	Microtubule Binding (GO:0008017)	0.005412
5	Alpha-Tubulin Binding (GO:0043014)	0.005618

Index	GO Biological Process 2023	P-value
1	Positive Regulation Of Transporter Activity (GO:0032411)	8.673E-06
2	Establishment Of Spindle Orientation (GO:0051294)	0.0001492
3	Neuron Projection Morphogenesis (GO:0048812)	0.0009083
4	Long-Term Memory (GO:0007616)	0.001571
5	Vesicle Transport Along Microtubule (GO:0047496)	0.001903

CP

Index	GO Molecular Function 2023	P-value
1	Protein Phosphatase 2A Binding (GO:0051721)	0.007033
2	Histone Deacetylase Binding (GO:0042826)	0.01734
3	Calcium Channel Regulator Activity (GO:0005246)	0.01761
4	Protein Phosphatase Binding (GO:0019903)	0.02563
5	arachidonate-CoA Ligase Activity (GO:0047676)	0.0272

Index	GO Biological Process 2023	P-value
1	DNA Deamination (GO:0045006)	0.001908
2	Positive Regulation Of Mitotic Cell Cycle Phase Transition (GO:1901992)	0.005059
3	Positive Regulation Of G2/M Transition Of Mitotic Cell Cycle (GO:0010971)	0.00588
4	Positive Regulation Of Cell Cycle G2/M Phase Transition (GO:1902751)	0.007645
5	Secondary Alcohol Biosynthetic Process (GO:1902653)	0.007645

Supplementary Table 2 - Demography of the human post-mortem cohort assayed by GeoMx.

Group	Gender (M/F)	Age at death (years)	Post-mortem delay (hours)	DV200
Aged Healthy Controls	3/7	92.0 (5.75)	25 (8.25)	28.9 (16.4)
Early PD	5/3	73.5 (12)**	17.5 (13.5)*	28.3 (9.10)

Values are presented as median (IQR). The comparison of Age at death, Post-mortem delay and DV200 between groups was made using the Welch Two Sample t-test. The comparison of gender between groups was made using chi-square test. * $P < 0.05$, ** $P < 0.001$.
